# Gene Set-Based Module Discovery Decodes *cis*-Regulatory Codes Governing Diverse Gene Expression across Human Multiple Tissues

**DOI:** 10.1371/journal.pone.0010910

**Published:** 2010-06-09

**Authors:** Atsushi Niida, Seiya Imoto, Rui Yamaguchi, Masao Nagasaki, Satoru Miyano

**Affiliations:** Human Genome Center, Institute of Medical Science, University of Tokyo, Tokyo, Japan; Fondazione Telethon, Italy

## Abstract

Decoding transcriptional programs governing transcriptomic diversity across human multiple tissues is a major challenge in bioinformatics. To address this problem, a number of computational methods have focused on *cis*-regulatory codes driving overexpression or underexpression in a single tissue as compared to others. On the other hand, we recently proposed a different approach to mine *cis*-regulatory codes: starting from gene sets sharing common *cis*-regulatory motifs, the method screens for expression modules based on expression coherence. However, both approaches seem to be insufficient to capture transcriptional programs that control gene expression in a subset of all samples. Especially, this limitation would be serious when analyzing multiple tissue data. To overcome this limitation, we developed a new module discovery method termed BEEM (Biclusering-based Extraction of Expression Modules) in order to discover expression modules that are functional in a subset of tissues. We showed that, when applied to expression profiles of human multiple tissues, BEEM finds expression modules missed by two existing approaches that are based on the coherent expression and the single tissue-specific differential expression. From the BEEM results, we obtained new insights into transcriptional programs controlling transcriptomic diversity across various types of tissues. This study introduces BEEM as a powerful tool for decoding regulatory programs from a compendium of gene expression profiles.

## Introduction

Predicting 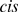-regulatory codes governing transcriptional programs in a specific type of cells has been intensively investigated by combining microarray gene expression data with 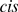-regulatory sequences or related information like ChIP-chip experiments. Recently, several attempts have been done for identifying tissue-specific 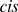-regulatory codes by applying these methods to microarray data of human multiple tissues in order to understand their diversity [1]–[5]. However, since these methods only consider comparing overexpression and underexpression in a single tissue with those in the other tissues, single-tissue specific 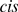-regulatory codes could only be found; 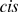-regulatory codes existing across several tissues were possibly failed to be discovered.

In this paper, to analyze multiple tissue data more efficiently, we propose a computational method for discovering such 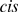-regulatory codes existing in the subset of samples by extending our previously proposed method called EEM (Extraction of Expression Modules) [6], [7]. EEM combines various kinds of biological information represented as gene sets with microarray data to find coherent genes as functional expression modules. An input gene set is prepared by collecting genes, which are considered to constitute an expression module, based on prior biological knowledge, e.g., a TF binding motif. For each gene set, EEM tests whether it harbors a coherently expressed subset; the coherent subset is then extracted as an expression module if it is significant. Although we previously showed that EEM is applicable a wide range of transcriptome data, EEM also has a limitation. Since EEM assumes that module genes, i.e., genes belonging to the same expression module, behave similarly across all samples, EEM potentially fails to identify an expression module whose module genes exhibit coherent expression patterns over only a subset of samples, i.e., *sample subgroup-specific expression module*. Especially, this problem should be serious when analyzing a diverse gene expression data set like a multiple tissue data set.

To overcome this limitation, we have developed an extended version of EEM termed BEEM (Biclustering-based EEM), which employs a biclustering algorithm to unravel sample subgroup-specific expression modules. The biclustering algorithm performs simultaneous clustering of rows and columns of a gene expression matrix to identify biclusters, i.e., a subset of genes that exhibit similar expression patterns across a subset of samples, and *vice versa*. While ordinary one-dimensional clustering assumes expression coherence across all samples as EEM does, a number of biclustering methods have been introduced for expression data analysis to relax this assumption [8]–[10].

In this study, we apply BEEM to an expression data set from human multiple tissues [1]. By targeting transcriptional modes that previous approaches cannot cover, BEEM successfully identified 11 sample subgroup-specific expression modules with their regulatory motifs. We establish a new module discovery method, BEEM, which would be suitable for analysis of heterogeneous transcriptome data.

## Results

### BEEM Algorithm

EEM, an existing method, assumes that module genes behave similarly across all samples in the expression profile data. This assumption is reasonable when the data were derived from focused experiments and the profiled transcriptome has less diversity. However, if the data contain heterogeneous transcriptome from a broad range of samples, an alternative would be more reasonable; module genes are assumed to be co-regulated in only a subset of samples. Based on this alternative assumption, BEEM employs a novel statistic termed the *BEEM statistic* to evaluate functionality of an input gene sets as an expression module.

The BEEM statistic is calculated using a biclustering algorithm, ISA (Iterative Signature Algorithm) [10]. ISA takes as input an expression matrix and a seed gene set, and searches for a bicluster; ISA assumes a bicluster as a subset of genes which exhibits higher or lower expression than a predefined threshold across a subset of samples, and *vice versa*. Starting with a seed gene set, all samples are scored by average expression values for this gene set and those samples are chosen for which the score exceeds a predefined threshold (the 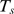 parameter, see Materials and Methods). In the same way, all genes are scored regarding the selected samples and a new set of genes is selected based on another threshold (the 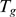 parameter, see Materials and Methods). The entire procedure is repeated until the set of genes does not change anymore. Although another biclustering algorithm can be employed in BEEM, we chose ISA because it starts a search from a seed set as well as EEM does, and we can easily combine ISA and the EEM approach. Another important advantage is that ISA is significantly fast compared to other biclustering algorithms [11], and tolerable for screening hundreds of gene sets.

**Figure 1:**
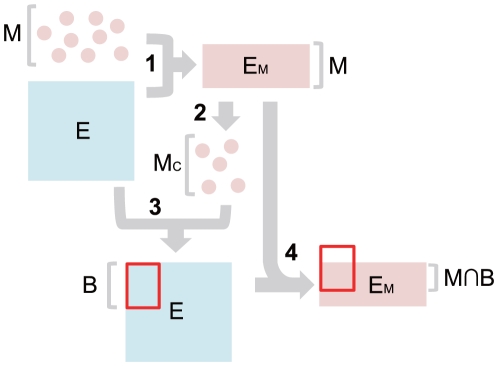
The biclustering pipeline in BEEM. 1) From the input expression matrix 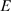, a submatrix 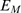 is extracted, which corresponds to the expression profiles of an input gene set 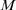. 2) EEM is then applied to 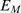 in order to obtain 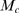, a maximal-sized coherent gene subset of 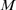, without statistical evaluation of its size. 3) Using 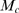 as a seed gene set, we apply ISA to 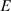, to obtain a bicluster (denoted by the red rectangle) and its biclusted gene set 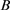. 4) Finally, the intersection of 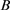 and 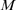 is obtained and used for statistical evaluation of the biclustered gene set in 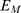.

Let 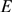 denote an input expression matrix whose rows and columns index genes and samples, respectively. We then define 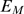, a submatrix of 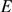 whose rows correspond to expression profiles of the members of an input gene set 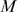. Employing ISA, BEEM tests whether 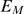 harbors any significantly large bicluster. To prepare a seed gene set for ISA, BEEM first extracts a maximal-sized coherent subset in 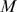, denoted as 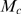, based on the EEM algorithm. Note that we do not care whether 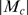 is significant; hence, the possibility is opened that BEEM captures gene sets that EEM misses. Next, using 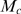 as the seed set, BEEM finds a bicluster from 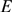. Let 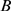 denote a gene set that constitutes the bicluster (or simply a *biclustered gene set* ) in 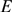. (Note that 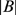 is constant when the 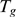 parameter is fixed; see below). The intersection 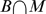 then constitutes a biclustered gene set in 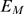 and we define 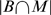 as the BEEM statistic. A series of these steps are illustrated in Figure 1.

It should be noted that BEEM extracts a bicluster from 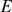 (not 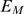). The reasons why we take this indirect strategy are: 1) Applying ISA to a relatively small matrix, 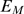 in our case, leads to unstable solutions and iterative calculation often does not converge. 2) When we apply ISA to 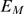 with equal-sized input gene sets, the size of extracted biclustered gene sets are constant. Therefore, in this case, the size of the biclustered gene sets then cannot be used as a measure of strength of the association between the input gene set 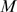 and the identified bicluster in 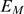. Hence, we decided to apply ISA to 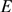 for controlling the size of the biclustered gene set, i.e., 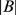; 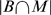 reflects strength of the association between 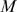 and the identified bicluster in 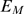.

BEEM calculates a p-value for representing the statistical significance of the BEEM statistic, 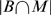; if the p-value is smaller than the prespecified cutoff value, we assume that 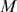 harbors an expression module and extracts 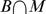 as the expression module. Note that results of BEEM depend on combinations of two parameter values, 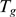 and 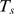 and the type of targeted biclusters, i.e., upregulated and downregulated biclusters. Therefore, for each gene set, we run BEEM with various settings and chose the result which scores the most significant p-value. The final p-value is reported after correcting multiplicity of the hypothesis testings. In Materials and Methods, we describe the ISA algorithm used in BEEM and the detail of p-value calculation for the BEEM statistic.

### Comparison with Other Methods

To characterize the performance of BEEM, we compared the performance of BEEM with those of two other methods based on different approaches. One of the two methods is EEM, which targets expression coherence across all samples. The other method targets single sample-specific expression. Although a number of methods taking the single sample-targeting approach have been proposed, we focused on a hypergeometric test-based method by Segal *et al.* [12]. Unlike BEEM and EEM, since Segal's method tests over and underexpression of a gene set in each sample, it does not explicitly assign a single p-value to the gene set. To make comparison easier, we thus reformulated Segal's method by combining statistical meta-analysis so that each gene set can obtain a single p-value, which is used for testing whether the gene set is over or underexpressd in any samples. As a representative of single sample-targeting methods, we employed this reformulated method termed SSA (Single Sample Analysis) for the benchmark test.

#### Performance evaluation on simulated data

First, we performed a benchmark test using simulated data. A set of simulated data consists of an expression matrix and a gene set library containing positive and negative gene sets. We assume that the expression matrix harbors a number of expression modules and a positive gene set in the gene set library has a significant overlap with any of the expression modules. To generate the input data set, we used different models assuming different types of expression modules described below. Since each model has arbitrary parameters, we tested a number of data sets using several different parameter settings. By applying BEEM, EEM and SSA to each of the simulated input data sets, we calculated sensitivities and false positive ratios over the whole range of significance cutoffs, and computed the Area Under the receiver operating characteristic Curves (AUCs). Since the AUC assesses the overall discriminative ability of the methods at determining whether a given gene-set is associated with an expression module, we assume it as a measure of the performance in this benchmark test. To reduce sampling variance, the results were obtained by averaging 20 Monte Carlo trials.

**Figure 2:**
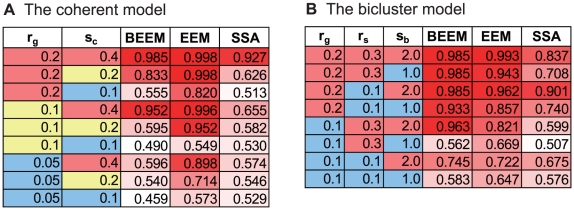
Comparison of AUCs among BEEM, EEM and SSA using simulated data. The AUCs were computed by applying the three methods to simulated data generated from two types of models. For each of the two simulation models, various patterns of parameter settings were examined.

The results can be summarized as follows: The first model, coherent model, assumes that genes that belong to the same expression module are coherently expressed across all samples. Such coherent expression modules should be efficiently extracted by EEM. Expectedly, EEM scores the best performance among the three methods, while BEEM performs substantially well, as compared to SSA (Figure 2A). In the other model, bicluster model, module genes are assumed to be overexpressed in a subset of samples; since BEEM was developed to target this type of expression modules, BEEM shows the best performance for this model. EEM also performs comparably well, but SSA performs worst again for this model (Figure 2B). Taken together, our results suggest that BEEM successfully captures sample subgroup-specific expression modules, while it also shows good performance to some degree for coherent expression modules, which are most efficiently captured by EEM.

#### Performance evaluation on real data

We performed another benchmark test using real biological data. The input data also include two types of information: expression data sets and gene set libraries. The expression data sets were obtained from two sources. One is a breast cancer data set, to which we applied EEM in our previous study [6], [13]. The other is a human multiple tissue data set, which has been subjected to a number of single sample-targeting methods [1]–[3], [5]. In addition to these expression data sets, we also prepared two permutated expression data sets by randomly shuffling their gene labels; we used them to evaluate the false positive rates of the three methods, assuming that they follow null hypotheses. As input gene sets, we prepared two types of gene set library: TF binding motif gene sets and curated gene sets. Based on TRANSFAC data [14], 199 TF binding motif gene sets are predicted to contain genes that share common TF binding motifs in their promoters; they can be used to analyze transcriptional programs. On the other hand, the curated gene set library contains miscellaneous 1892 gene sets extracted from original literature [15].

**Figure 3:**
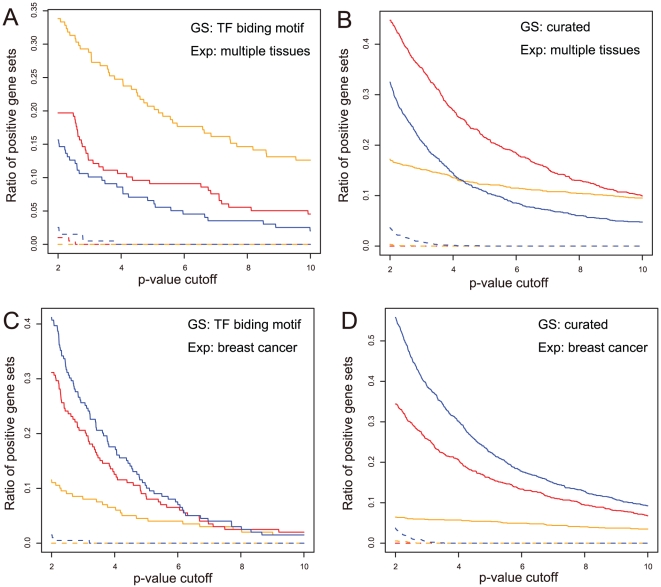
Comparison of performance among BEEM, EEM and SSA using real data. While changing p-value cutoff values, which are given in minus log scale, ratios of positive gene sets detected by BEEM, EEM and SSA were plotted for the 4 combinations of the input data: the TF binding motif gene sets and breast cancer expression data set (A); The TF curated gene sets and breast cancer expression data set (B); the TF binding motif gene sets and multiple tissue expression data set (C); the curated gene sets and multiple tissue expression data set (D). Red, blue and yellow lines indicate performance of BEEM, EEM and SSA, respectively. Dashed lines represents results obtained from null expression data sets whose gene labels were randomly permutated.

We applied BEEM, EEM and SSA to every combination of input data sets; i.e., we performed 24 analyses using three methods, four expression data set, and two gene set libraries. For each analysis, we counted positive gene sets whose p-values are smaller than a cutoff value. Note that we tested wide range of cutoff value for showing the power and false positive rate of each method. Figure 3 shows the ratios of positive genes set for given p-value cutoffs (See also Tables S1, S2, S3, S4 in Supplemental Files for raw p-values). First, we evaluated the false positive rates of the three methods using the permuted expression data described by the dashed lines. Although the number of false positives of EEM is slightly larger than those of others, the false positive rates of the three methods are satisfactorily controlled. We then compared the performance by testing which method retrieves more positive gene sets for a given significance level, i.e., p-value cutoff. When comparing BEEM to EEM, we found that BEEM outperforms EEM for the multiple tissue data sets, but EEM identified more positive gene sets than BEEM in the breast cancer data set. This result was observed for both of the two gene set libraries and presumably reflects the properties of the two expression profiles. The breast cancer data set obtained from tumors of single tissue origin should have relatively homogenous transcriptomes, and give a better fit to the coherent model shown in the simulated data test. On the other hand, the multiple tissue data set from various types of tissues seems to have more heterogeneous transcriptomes, and closes to the bicluster model.

**Figure 4:**
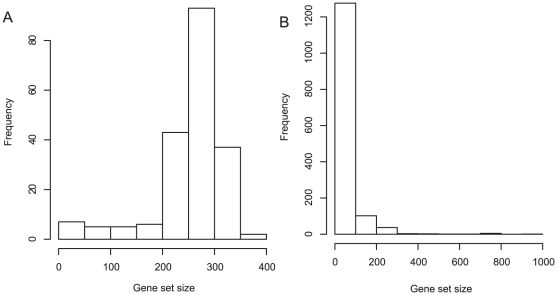
Distributions of the size of input gene sets. (A) TF binding motif gene set library and (B) curated gene set library.

**Figure 5:**
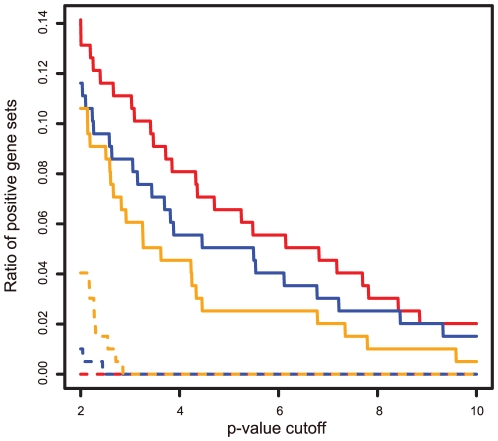
Comparison of performance using the downsized TF binding motif gene sets. Ratios of positive gene sets detected by BEEM, EEM and SSA were measured using the 50% downsized TF binding motif gene sets and the multiple tissue expression data set. Red, blue and yellow lines indicate the results of BEEM, EEM and SSA, respectively. Dashed lines represent the results obtained from null expression data sets whose gene labels were randomly permutated.

Next, we focused on the comparison between BEEM and SSA. When applied to the breast cancer data set, SSA shows very poor performance, as compared to BEEM and EEM. This result seems to be natural by considering a homogenous property of the breast cancer data. For the multiple tissue data set, the performance of BEEM depends on the type of input gene set libraries; SSA works better for the TF binding motif gene set library while BEEM works better for the curated gene set library. We presume the reason is that the two gene set libraries have different distribution of gene set sizes (Figure 4). We observed that the distribution of the number of genes contained by each of the TF binding motif gene sets is nearly bell-shape and has a peak in the range from 250 to 300. On the other hand, the distribution of the curated gene sets is skewed and the sizes of almost all gene sets are smaller than 100. Based on this observation, we hypothesized that the performance of SSA depends strongly on the sizes of input gene sets. To validate this hypothesis, we focused on the distribution of the sizes of positive gene sets retrieved by each method, especially the result for the multiple tissue expression data set and curated gene set library (because, for this input combination, all the three methods have a number of positive gene sets of diverse sizes). After partitioning gene set size to 6 intervals, for each method, we calculated frequency of positive gene sets contained in each interval. Then, by dividing frequency of positive gene sets by that of input gene set, we calculated relative performance of each method in each interval of gene set size (Table 1). We found that, although all the methods expectedly show higher performance for lager gene sets, SSA shows stronger dependency on gene set size than BEEM and EEM. Especially, in the interval from 200 to 400 where the TF binding motif gene set library has the peak in the size distribution, the performance of SSA is twice as high as those of BEEM and EEM. This observation suggests that the dependency on the size of the gene set is a reason why SSA shows higher performance for the TF binding motif gene set library. To test this hypothesis more directly, we prepared downsized TF binding motif gene sets. A downsized gene set was generated by randomly sampling genes from an original gene set so that its size is equal to the half of the original size. By applying BEEM, EEM and SSA to the downsized TF binding gene sets, we found that the performance of SSA get worse, while BEEM and EEM kept their capability (Figure 5). Taken together, our data suggest that the performance of SSA for the TF binding motif gene set library is artifactually enhanced by its gene set-size dependent property.

**Table 1:** Relative performance in each size interval of gene sets.

size	SSA	BEEM	EEM
0–25	0.031022	0.354167	0.418033
25–50	0.713579	1.124139	1.173354
50–100	2.458843	1.692075	1.500631
100–200	3.678832	2.598485	2.295082
200–400	4.429927	2.298864	2.633607
400–1000	8.338686	4.327273	3.511475

**Figure 6:**
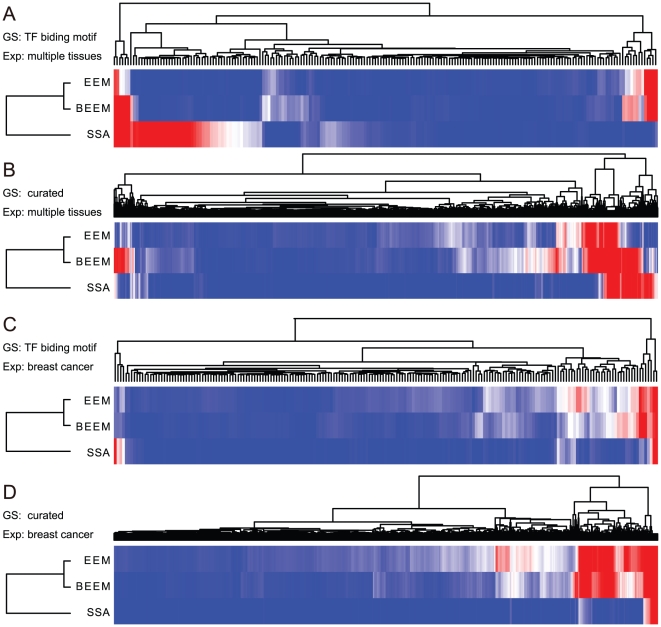
Comparison of p-value distributions among BEEM, EEM and SSA. Minus log-scaled p-values of each gene set calculated by BEEM, EEM and SSA were visualized using heatmaps after the values that exceed 10 were set to 10 (more significant: red, less significant: blue). The 4 heatmaps corresponds to the 4 combinations of the input data: the TF binding motif gene sets and breast cancer expression data set (A); The TF curated gene sets and breast cancer expression data set (B); the TF binding motif gene sets and multiple tissue expression data set (C); the curated gene sets and multiple tissue expression data set (D).

**Figure 7:**
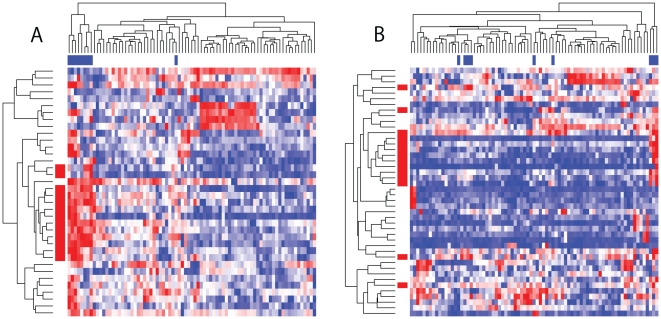
Expression profiles of two gene sets that are significant only in BEEM analysis. A) Expression: multiple tissues, Gene set: TAVOR_CEBP_UP in the cutated library, and B) Expression: multiple tissues, Gene set: LEE_CIP_DN in the curated library. The heatmap shows 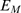 (increased expression: red, decreased expression: blue). Rows and columns index genes and samples, respectively. Red bars attached to rows represent biclustered gene sets (corresponding to 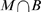), while blue bars attached to columns represent biclustered sample sets. The p-values assigned to these expression profiles by BEEM, EEM and SSA are: A) 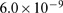, 0.083 and 0.26; and B) 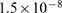, 0.14 and 0.26, respectively.

Finally, we examined differences of the positive gene sets retrieved by the three methods. For the four analyses of different combinations of input gene set library and expression data set, we drew the heatmaps of p-values of all gene sets obtained by the three methods (Figure 6). They show that positive gene sets detected by the three methods are not identical but partially overlapping. Note that, although EEM and BEEM produce relatively similar results, positive gene sets by BEEM roughly comprehend those by EEM in the multiple tissue data set, but opposite in the breast cancer data set. This result suggests the differences between BEEM and EEM for the expression data sets with various sample diversity. Although SSA behaves differently from two other methods, it produces results more similar to BEEM than EEM. This observation seems to reflect similarity of two approaches. Especially, by focusing on the results for the multiple tissue data set, we found that the BEEM approach is positioned between the two others. BEEM extracted not only all of positive gene sets by both EEM and SSA, but also gene sets that the two other methods could not find. Figure 7 shows two bicluster structures successfully detected only by BEEM. Collectively, our benchmark test using real data demonstrates that BEEM successfully targets not only transcriptional programs which are covered by either of EEM and SSA, but also novel types of transcriptional programs which have not been covered by either of the two previous approaches.

### Expression Module Discovery in the Multiple Tissue Transcriptomes

**Figure 8:**
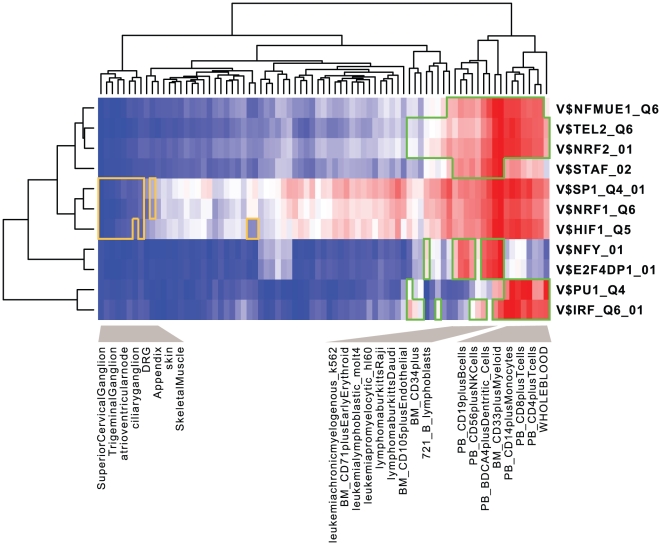
Activity profiles of expression modules in the multiple tissues data set. For 11 expression modules identified by BEEM, activity profiles were calculated, subjected to hierarchical clustering, and displayed as a heat map (increased activity: red, decreased activity: blue). Green and yellow polygons indicate samples that constitute up and down-regulated bicluster identified by ISA.

**Figure 9:**
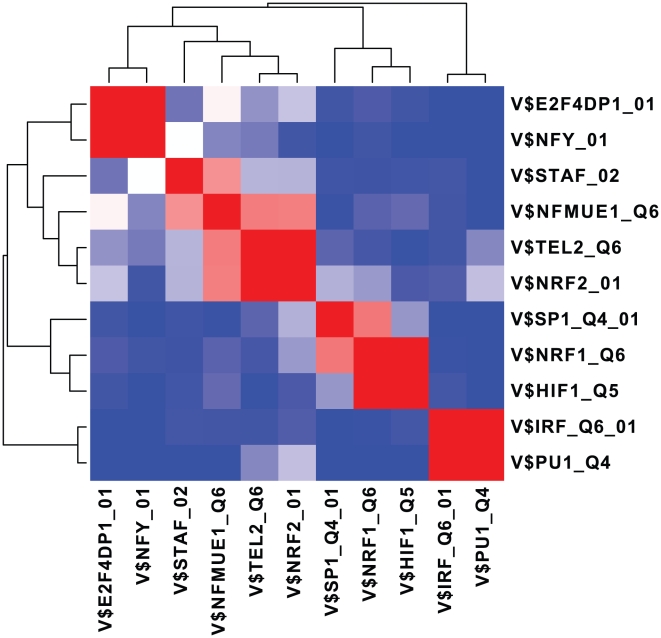
A Overlaps of expression modules in the multiple tissues data set. Overlaps among 11 expression modules were tested by hypergeometric tests, and the p-values in minus log scale were visualized as a clustered symmetric matrix. The values that exceed 10 were set to 10. Red and blue indicates more and less overlaps, respectively.

In our previous study, we showed that EEM successfully decodes transcriptional programs in breast cancer cells [6]. Similarly to EEM, when given a TF binding motif gene set, BEEM predicts genes under a common *cis*-regulatory code as an expression module; Furthermore, the extracted module information can be used to inspect the upstream transcriptional program. In this section, from the results of BEEM analysis, we tried to obtain new insights into *cis*-regulatory codes governing transcriptomic diversity across various types of human tissues. We obtained positive TF binding motif gene sets using the cutoff of 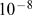 and 11 significant expression modules are extracted (Table 2 and Table S5). Compared to the EEM and SSA results, BEEM assigns smaller p-values to most of the 11 expression modules. Intriguingly, most of the expression modules score significant p-values in either of EEM and SSA. This observation suggests that BEEM can detect two different types of modules targeted by the other two methods. Some expression modules, however, score significant p-values only in BEEM, demonstrating that BEEM captures transcriptional programs that the other methods fail to detect. Since many of them are enriched for specific GO terms, BEEM successfully identified functional units in the transcriptome. We also drew the activity profile of each expression module, which is defined as the mean values of the expression profiles of the module genes: the heat map in Figure 8 shows in which tissues each expression module is up or down-regulated. From the heat map, we found that 11 expression modules are divided into four distinct clusters. Moreover, we tested overlaps between expression modules; the p-value matrix in Figure 9 shows that, in each of the four clusters, the expression modules share a significantly large number of genes while there are little overlaps between expression modules that belong to different clusters. These observations suggest that they are not independent expression modules, but might be subsets of the same large expression module regulated by multiple interacting motifs.

**Table 2:** Expression modules in the multiple tissues data set.

TRANSFAC ID	BEEM p-value*	EEM p-value*	SSA p-value*	enriched GO**
V$E2F4DP1_01	28.63	8.84	Inf	DNA replication (13.66)
V$NFMUE1_Q6	18.81	28.57	0.77	RNA binding (8.00)
V$NRF2_01	16.77	16.72	0.027	structual component of ribosome (10.50)
V$NFY_01	16.53	4.15	12.88	cell cycle (9.61)
V$NRF1_Q6	13.65	10.27	2.25	-
V$TEL2_Q6	13.33	10.02	0.0016	-
V$PU1_Q4	11.49	3.78	Inf	defense response (5.18)
V$HIF1_Q5	8.75	6.69	0.019	-
V$STAF_02	8.46	4.94	0.040	macromolecular complex (4.97)
V$IRF_Q6_01	8.41	0.12	8.26	immune respose (7.34)
V$SP1_Q4_01	8.20	4.08	0.0021	-

*p-values are shown in minus log scale.

**p-values in minus log scale are given in the parentheses.

The composition of the four clusters is given as follows: “V$E2F4DP1_01 and V$NFY_01”; “V$PU1_Q4 and V$IRF_Q6_01”; “V$NFMUE1_Q6, V$NRF2_01, V$TEL2_Q6 and V$STAF_02”; “V$NRF1_Q6, V$HIF1_Q5 and V$SP1_Q4”. Note that we refer to expression modules using TRANSFAC IDs of their regulatory motifs. The activity profiles show that the expression modules in the first cluster, V$E2F4DP1_01 and V$NFY_01, are upregulated in a sample subgroup enriched for bone marrow, lymphoma and leukemia cells. These expression modules regulated by E2F and NFY harbor many cell cycle-related genes, presumably reflecting that cells are actively proliferated in these tissues. The expression modules in the second cluster, V$PU1_Q4 and V$IRF_Q6_01, are activated in a sample subgroup enriched for immunes cells extracted from peripheral blood; they contain many immune-related genes, suggesting PU1 and IRF cooperatively regulate immune systems in blood cells. The activity profiles show that the expression modules in the third cluster, V$NFMUE1_Q6, V$NRF2_01, V$TEL2_Q6 and V$STAF_02, are upregulated in tissues where the former two expression module clusters are activated, while the GO term analysis shows they share ribosomal components. By combining these different types of information, we speculate that these tissues also have active translational systems upregulated by NFMUE1, NRF2, TEL2 and STAF. The expression modules in the fourth cluster, V$NRF1_Q6, V$HIF1_Q5 and V$SP1_Q4, are downregulated in sample subgroups containing ganglions; however, we could not find any significant GO terms, and their function remains to be elucidated. SSA assigns significant p-values to the expression modules in the first and second clusters scores, presumably reflecting that they are specifically expressed in a small number of tissues. On the other hand, the expression modules in the third and fourth clusters do not have significant p-values in SSA. Although some of them also have significant p-values in EEM, the others are only marginally significant in EEM. This result demonstrates that, from the multiple tissue transcriptomes, BEEM successfully discovered expression modules that cannot be captured by the traditional approaches.

## Discussion

Here, we have introduced a new module discovery method, BEEM, to analyze sample subgroup-specific transcriptional programs which are functional only in subgroups of samples. We compared BEEM to two other methods, EEM and SSA, which target coherent expression and single sample specific-expression, respectively. We found that BEEM and EEM produce relatively similar results, but their performances seem to be different depending on heterogeneity of input transcriptome data: BEEM works better for analyzing more heterogeneous data like the multiple tissue data set.

**Figure 10:**
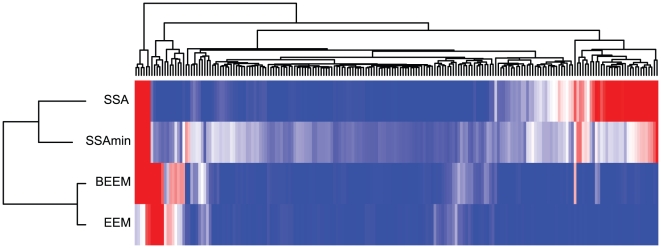
Comparison of the minimums of pre-combined SSA p-values to p-values by other methods. SSAmin presents the minimums of pre-combined p-values in SSA. For SSAmin and other methods, minus log-scaled p-values were calculated using the TF binding motif gene set and multiple tissue expression data set, and were visualized as a heatmap.

Although SSA performs well for analysis of tissue-specific transcriptional programs, performance of SSA is highly dependent on the size of input gene sets; i.e., BEEM seems superior to SSA for analysis of gene sets of relatively small size, typically smaller than 50. For this strong dependency of SSA on input gene set size, one possible reason can be provided; it is because SSA combines p-values for individual samples by Fisher's method. Note that the combined p-value could be significant even when none of the individual hypergeometric p-values are clearly significant. For a larger sized input gene set, this fact should more strongly affect the SSA results because it is more probable that different subsets of the input gene set are over or underexpressed in different samples. We actually found that, for most of positive gene sets only found by SSA but not by BEEM, the minimums of their original (pre-combined) p-values are only marginally significant (Figure 10), suggesting that their expressions are not specifically regulated in any tissues. Taking into account this observation, apparently better performance of SSA for gene set of large size does not lead to more biologically meaningful findings; on the other hand, BEEM can present more interpretable results as expression modules as discussed below.

More importantly, BEEM covers a broad range of transcriptional modes positioned between two different modes targeted by EEM and SSA; the biclustering algorithm enables BEEM to capture expression modules with intermediate properties, which are missed by two types of previous approaches. However, it should be noted that BEEM also failed to detect some expression modules, which the other methods could capture. For example, although it is known that HNF1 regulates expression of liver-specific genes [3], BEEM does not assign a significant p-values to the HINF1 expression module in the multiple tissue data set, while SSA assigns a significant p-value. This is because the sample subgroup where the module genes are expressed is too small to be detected by the biclustering algorithm. We expect that combining our proposed method with conventional approaches leads to more comprehensive discovery of transcriptional programs.

We should also mention another notable advantage of BEEM. Application of BEEM to the multiple tissues expression data set discovered of 11 regulatory motifs that regulate the diverse transcriptomes. Similarly to EEM, BEEM produces information about many regulatory links between TF binding motifs and their target genes as expression modules. We can have information about in which tissues each motif is functional from activity profiles of expression modules. By clustering the obtained expression modules based on the similarity of the activity profiles and module overlap, we predicted interacting pairs of TF binding motifs. Cellular function of each TF binding motif was also inferred from the GO terms enriched in their target genes. A series of these post-BEEM analyses generated highly interpretable biological knowledge, demonstrating the power of our module-based approach. Taken together, this study has established BEEM as a powerful alternative for decoding regulatory programs from a compendium of gene expression profiles.

## Materials and Methods

### ISA

Given a seed gene set and the values of parameters 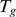 and 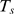, ISA searches for a bicluster in an 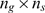 matrix 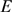, whose 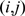-th element 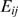 represents the expression value of the 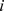-th of 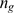 genes and the 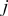-th of 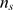 samples. For 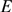, we prepared two types of normalized matrices, 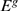 and 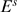. Each column vector of 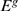 and each row vector of 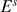 were normalized so that the mean is equal to 0 and the variance is equal to 1 (i.e., 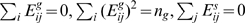, and 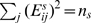).

A bicluster can be specified by a binary sample vector 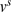 of length 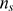 and a binary gene vector 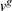 of length 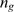, where non-zero entries in the vectors indicate samples/genes that belong to the bicluster. After 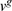 is initialized so that non-zero entries indicate genes in the given seed gene set, ISA iteratively updates 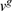 and 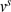. First ISA calculates a sample-score vector 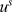 which scores each sample according to how much the non-zero genes in 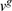 is upregulated:
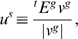
where 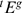 is the transpose of 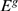, and 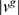 is the number of the non-zero entries in 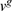. Next, ISA uptates the sample vector 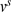, which scores whether the elements of 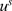 that are above a threshold 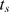:
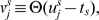
where 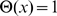 for 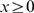 and 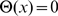 for 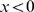. Although 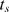 is a fixed parameter in the original paper [10], we set 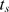 to the 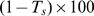-th percentile of 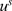. Similarly to 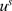, the gene-score vector 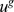 measures how much each gene is upregulated under the non-zero samples defined in 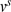:
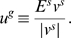
Based on 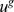, 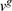 is then updated for an input of the next iteration:
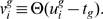
Similarly to 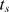, we set 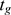 to the 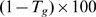-th percentile of 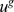.

These steps are repeatedly performed untile the gene vector 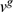 does not change anymore. Non-zero elements in 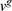 and 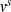 then specify an upregulated bicluster, which consists of approximately 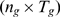 genes and 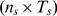 samples. By inverting signs the normalized matrices (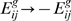, 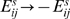) prior to the calculation, ISA can also target downregulated biclusters.

### Calculation of p-value for the BEEM Statistic

**Figure 11:**
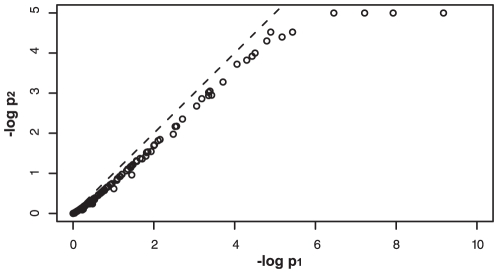
Comparison of 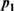 and 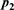. 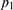
 and 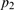 were plotted in log minus scale. they were calculated using the TF binding motif gene sets and multiple tissue expression data set with a parameter setting of 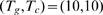. To calculate 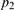, random samplings was performed 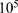 times and 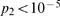 were plotted as 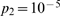. Similar results were also obtained for different inputs and parameter settings.

To calculate a p-value for the BEEM statistic, 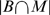, BEEM takes a three-step approach. First, we roughly calculated a p-value based on the hypergeometric distribution, which is popularly used to evaluate overlap between two gene sets [16]:
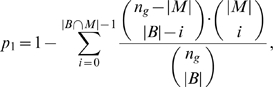
where 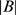 is the number of the genes that constitute the bicluster in the input expression matrix, 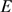. Note that 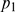 tends to be liberal, i.e., it tends to generate false positives as shown in Figure 11. It is possibly because, even if 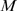 is a null gene set, it is associated with 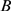 via 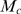 (Note that 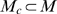 and 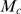 is also the seed gene for 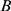). It is, however, reasonable to use a liberal p-value for the first step, because we want to remove the gene sets that are really insignificant in the first step. In the second step, we employ a computer intensive method to compute more accurate p-value and the first step contributes to reduce the computational time in the second step.

If 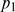 is smaller than a threshold (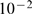 in this study), BEEM then calculates a more accurate p-value, 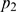, based on an empirical approach. An empirical null distribution for a BEEM statistic is produced by randomly sampling 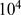 gene sets whose size is equal to that of the seed gene set, and calculating 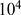 BEEM statistics following the null distribution. The p-value is then calculated as a ratio of null statistics which are larger than or equal to the BEEM statistic evaluated.

However, if it relies only on this empirical approach, it is impossible to calculate 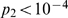. Of course, by increasing the number of the null statistics, we can have smaller p-values. However, it practically needs prohibitive computational time. To overcome this limitation, we extrapolate 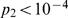 based on a relation between 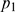 and 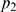, We found that, for the same expression matrix and fixed parameters, 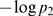 linearly correlates with 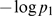 very well when 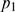 is small enough (Figure 11). Since BEEM is usually applied to a hundred of gene sets to screen for meaningful gene sets, we could obtain dozens of pairs of 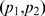 for gene sets which meet the criterion in the second step (i.e., 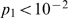). The missing 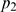
's that are smaller than 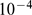 are predicted from the values of 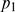 by the linear regression. i.e., 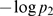 is the response variable and 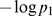 is the explanatory variable.

In ISA, the choices of 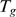 and 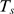 and the type of targeted bicluster are critical for obtaining the optimal bicluster associated with each seed gene set. Hence, we performed BEEM with nine combinations between 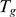 (0.05, 0.1, and 0.15) and 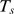 (0.1, 0.2 and 0.3). We also target two different types of bicluster: up and down-regulated. In total, we examine 18 settings and selected the best result which scores the minimum p-value. Since the best p-value, 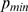, is liberal due to the multiplicity of the hypothesis testings, it should be corrected to obtain a final p-value 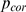 as follows:
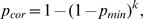
where 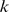 is the number of the examined settings (i.e., 18 in our case).

### Input Data for BEEM

#### Simulated Data

We simulated expression matrices and gene set libraries for the input data. We assumed that an expression matrix includes 4000 genes and 100 samples, and harbors a number of expression modules, each of which is associated with a subset of the 4000 genes. A gene set library is assumed to have positive gene sets, and negative gene sets. The positive gene sets were prepared so that they have significant overlaps with any of the expression modules, while the negative gene sets were randomly sampled from the 4000 genes.

To simulate expression matrices, we assumed two different models:

1. Coherent model.

We assumed that a 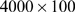 expression matrix has non-overlapping 20 modules, each of which consists of 200 module genes. For each module, we first chose one gene and generated its expression values across samples by the standard Gaussian distribution. That is, assuming that we chose gene 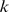, we have 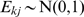 for 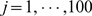. The other module genes were generated so that they gather around gene 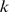. The expression value of gene 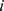 who is a member of the module generated from gene 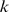 is generated by
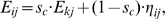
where 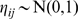 and 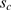 is a parameter specifying signal strength.

2. Bicluster model.

We assumed that a 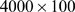 expression matrix has 50 modules, each of which consists of 200 module genes, and is allowed to overlap with each other. We randomly selected 200 genes from the 4000 genes to define module genes of each expression modules. Assuming each expression module as a biclustered gene set, we randomly chose 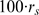 samples as a biclustered sample set for the module. Here, 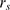 is a parameter specifying the ratio of the biclustered sample set. Let 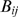 be an indicator variable, where 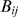 takes 1 if and only if the expression value of gene 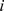 in sample 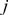, 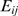, belongs to any of the defined bicluster, or 0 otherwise. We set
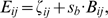
where 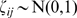 and 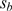 is a parameter specifying signal strength.

We simulated a gene set library including 10 positive and 10 negative gene sets, where each gene set includes 200 genes. A positive gene set includes 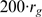 genes sampled from one expression module, and randomly sampled 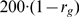 genes. Here, 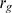 is a given parameter specifying the ratio of module genes in the positive gene set. On the other hand, a negative gene set was prepared by randomly sampling 200 genes.

#### Real Data

We downloaded two microarray data sets from the GEO database: a human breast cancer data set (GSE3494) [13] and a human multiple tissues data set (GSE1133) [1]. Absolute expression values of each data set were converted to the logarithmic scale and normalized so that the mean is equal to 0 and the variance is equal to 1 in each sample. The probe set IDs were converted to genes symbols. In cases that one gene symbol matches multiple probe set IDs, the probe set which shows the most variance across the samples was mapped to the gene. A variation filter was then applied to the data, and we obtained 8000 genes with the highest variance. The expression profiles of the 8000 genes were normalized across samples and subjected to the following analysis.

The TF binding motif gene set library was prepared as described in [6]. Briefly, we prepared human and mouse promoter sequences encompassing the 500 bp upstream and 100 bp downstream of the transcription start sites. We also prepared 199 PWMs from TRANSFAC 2009.1 [14], by applying motif clustering to all vertebrate TRANSFAC PWMs and removing redundant motifs. For each PWM, we scored every human and mouse promoter sequence based on maximum log odds scores, and obtained the average of human and mouse homolog promoter scores as the PWM score for each gene. We assumed genes which record the 5% highest PWM scores as seed gene sets sharing common TF binding motifs associated with the PWM. The curated gene set library including 1892 gene sets was downloaded from a gene set database, MSigDB [15]. As actual input to BEEM and other methods, we used the intersection of each gene set and 8000 genes in an input expression data set, after gene set for which the intersection was less than 10 were filtered out.

### EEM

The algorithm of EEM is described in detail in [6]. We used radius parameters of 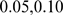 and 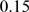 and calculated p-values using a recently developed efficient method (manuscript in preparation). The p-values corrected for multiple hypothesis testing were then obtained as described above.

### SSA

So far, a number of methods targeting gene sets differentially expressed in a single sample have been reported [2]–[5]. Among them, we focused on a simple but widely used approach based on the hypergeometric test. Since the approach introduced by Segal *et al.* does not explicitly assign a single p-value to an input gene set, we reformulated it and called it SSA (Single Sample Analysis).

First, SSA normalizes the input expression matrix 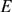 across samples to obtain 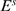. The 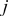-th column vector of 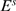, 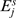 scores how much each gene is up or downregulated in the 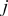-th sample, compared with an average across all samples. Based on values of 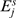, we can obtain the top 5% of the upregulated genes in the 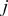-th sample, denoted as 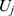. SSA tests overlap between the input gene set 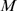 and 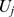 based on the hypergeometric test, and obtains a p-value, 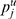, for upregulation of 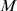 in the 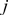-th sample. Similarly, SSA calculates a p-value, 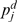, for downregulation of 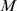 in the 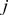-th sample. After 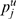 and 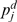 are calculated for all samples, we obtains a p-value vector of length 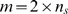, 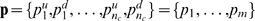. To assign a single p-value to 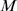, SSA converts 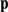 to the combined p-value by Fisher's method [17]. When up and downregulation across samples are independent, the overall significance of the 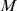 can be represented by a single statistic, whose p-value can be calculated from the chi-square distribution of 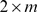 degrees of freedom:
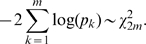
However, because gene expressions between samples are generally correlated, assumption of independence is not guaranteed; the tests based on the independence assumption could overestimate statistical significance, leading to more false positives. To correct the problem, we employed Brown's approximation for combining independent p-values [18]:
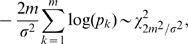
where 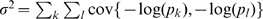. Note that 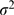 is unknown and needs to be estimated. We generated 1000 null gene sets whose sizes have the same distribution as the input gene sets, calculated **p** for each of them, and estimated 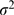 from the 1000 null p-value vectors.

### Expression Module Discovery in the Multiple Tissue Transcriptomes

By applying BEEM to the TF binding motif gene sets and multiple tissue expression data set, we assigned a p-value to each gene set. Using a cutoff value of 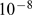, we obtained 16 significant gene sets out of 199 input gene sets, along with their 16 regulatory TF binding motifs. We found that the 16 TF binding motifs contain some cognate motifs which are similar to each other and seem to be bound by same the TF. To reduce the redundancy, we performed clustering. From the motif list in which the 16 motifs were sorted in ascending order of the p-values, we removed the 1st motif and, for each of the reminder, we calculated the KL distance from the first motif. If the distance is less than a cutoff value of 15 (we found that this cutoff value well discriminates between cognate and non-cognate motif pairs), we removed it from the sorted list and put it together with the 1st motif, assuming them as cognate motifs. This procedure was repeated until the sorted list got empty. We finally obtained 11 clusters of motifs and took the top scoring motif in each cluster as non-redundant TF binding motifs.

From the 11 gene sets having the non-redundant motifs, we extracted their subsets that constitute biclusters, i.e. 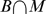, as expression modules. To predict functions of the expression modules, the GO enrichment tests were performed using the hypergeometric distribution [19]. To visualize the tissue specificity of the expression modules, the activity profile of each expression module was calculated by taking a mean of the expression profiles of the module genes, and presented as a heat map (Figure 8). Overlaps between each pair of the 11 expression modules were tested by hypergeometric tests. After the obtained p-values were transformed in minus log scale with base 10, the symmetric p-value matrix was visualized as a heat map (Figure 9).

## Supporting Information

Table S1 p-values by BEEM, EEM and SSA (GS:TF binding motif, Exp:multiple tissues).
(0.02 MB XLS)

Table S2 p-values by BEEM, EEM and SSA (GS:curated, Exp:multiple tissues).
(0.10 MB XLS)

Table S3 p-values by BEEM, EEM and SSA (GS:TF binding motif, Exp:breast cancer).
(0.02 MB XLS)

Table S4 p-values by BEEM, EEM and SSA (GS:TF curated, Exp:breast cancer).
(0.09 MB XLS)

Table S5 A result of BEEM (GS:TF binding motif, Exp:multiple tissues).
(0.01 MB XLS)

## References

[1] Su A, Wiltshire T, Batalov S, Lapp H, Ching K (2004). A gene atlas of the mouse and human protein-encoding transcriptomes.. Proc Natl Acad Sci U S A.

[2] Smith A, Sumazin P, Xuan Z, Zhang M (2006). DNA motifs in human and mouse proximal promoters predict tissue-specific expression.. Proc Natl Acad Sci U S A.

[3] Smith A, Sumazin P, Zhang M (2007). Tissue-specific regulatory elements in mammalian promoters.. Mol Syst Biol.

[4] Kim S, Kim Y (2006). Genome-wide prediction of transcriptional regulatory elements of human promoters using gene expression and promoter analysis data.. BMC Bioinformatics.

[5] Pennacchio L, Loots G, Nobrega M, Ovcharenko I (2007). Predicting tissue-specific enhancers in the human genome.. Genome Res.

[6] Niida A, Smith A, Imoto S, Aburatani H, Zhang M (2009). Gene set-based module discovery in the breast cancer transcriptome.. BMC Bioinformatics.

[7] Niida A, Imoto S, Nagasaki M, Yamaguchi R, Miyano S (2009). A novel meta-analysis approach of cancer transcriptomes reveals prevailing transcriptional networks in cancer cells.. Genome Informatics.

[8] Cheng Y, Church G (2000). Biclustering of expression data.. Proc Int Conf Intell Syst Mol Biol.

[9] Tanay A, Sharan R, Shamir R (2002). Discovering statistically significant biclusters in gene expression data.. Bioinformatics.

[10] Bergmann S, Ihmels J, Barkai N (2003). Iterative signature algorithm for the analysis of large-scale gene expression data.. Phys Rev E Stat Nonlin Soft Matter Phys.

[11] Prelic A, Bleuler S, Zimmermann P, Wille A, Bühlmann P (2006). A systematic comparison and evaluation of biclustering methods for gene expression data.. Bioinformatics.

[12] Segal E, Friedman N, Koller D, Regev A (2004). A module map showing conditional activity of expression modules in cancer.. Nat Genet.

[13] Miller L, Smeds J, George J, Vega V, Vergara L (2005). An expression signature for p53 status in human breast cancer predicts mutation status, transcriptional effects, and patient survival.. Proc Natl Acad Sci U S A.

[14] Matys V, Kel-Margoulis O, Fricke E, Liebich I, Land S (2006). Transfac and its module transcompel: transcriptional gene regulation in eukaryotes.. Nucleic Acids Res.

[15] Subramanian A, Tamayo P, Mootha V, Mukherjee S, Ebert B (2005). Gene set enrichment analysis: a knowledge-based approach for interpreting genome-wide expression profiles.. Proc Natl Acad Sci U S A.

[16] Jakt L, Cao L, Cheah K, Smith D (2001). Assessing clusters and motifs from gene expression data.. Genome Res.

[17] Fisher RS (1932). Statistical methods for research workers.

[18] Brown M (1975). A method for combining non-independent, one-sided tests of significance.. Biometrics.

[19] Rivals I, Personnaz L, Taing L, Potier M (2007). Enrichment or depletion of a go category within a class of genes: which test?. Bioinformatics.

